# Changes in Serum Levels and Gene Expression of *PGC-1α* in The Cardiac Muscle of Diabetic Rats: The Effect of Dichloroacetate and Endurance Training

**DOI:** 10.22074/cellj.2021.6942

**Published:** 2020-04-22

**Authors:** Hamed Rezaei Nasab, Abdolhamid Habibi, Masoud Nikbakht, Mohammad Rashno, Saeed Shakerian

**Affiliations:** 1. Department of Exercise Physiology, Faculty of Sport Sciences, Shahid Chamran University of Ahvaz, Ahvaz, Iran; 2. Department of Immunology, Faculty of Medicine, Ahvaz Jundishapur University of Medical Sciences, Ahvaz, Iran; 3. Cellular and Molecular Research Center, Ahvaz Jundishapur University of Medical Sciences, Ahvaz, Iran

**Keywords:** Endurance Training, Diabetes, Dichloroacetate, Mitochondrial Biogenesis

## Abstract

**Objective:**

Physical activity leads to changes in the level of gene expression in different kinds of cells, including changes in mitochondrial biogenesis in the myocardium in diabetic patients. Peroxisome proliferator-activated receptor γ coactivator 1α (PGC-1α) is a gene that plays an important role in regulating mitochondrial biogenesis. The purpose of this study was to investigate changes in serum levels and cardiac muscle expression of *PGC-1α* in diabetic rats in response to the administration of dichloroacetate (DCA) and endurance training.

**Materials and Methods:**

In this experimental study, 64 male Wistar rats were selected and randomly divided into eight groups after induction of diabetes with streptozotocin (STZ). The endurance training protocol was performed on a treadmill for 6 weeks. Intraperitoneal injection of DCA of 50 mg/ kg body weight was used for the inhibition of Pyruvate Dehydrogenase Kinase 4 (PDK4) in the myocardium. Gene expression were measured using real-time polymerase chain reaction (PCR). One-way ANOVA and Tukey’s test were used to statistically analyze the data.

**Results:**

The results of the study showed that PDK4 gene expression in the endurance training group, diabetes+endurance training group, diabetes+endurance training+DCA group and endurance training+DCA group was higher compared to the control group. Expression of PGC-1α was higher in the endurance training group compared to the control group but was lower compared to the control group in diabetes+endurance training+DCA group and diabetes+DCA group (P<0.05).

**Conclusion:**

Considering that *PGC-1α* plays an important role in mitochondrial biogenesis, it is likely that by inhibiting PDK4 and subsequently controlling oxidation of fatty acid (FA) in the heart tissue, oxidative stress in the heart tissue of diabetic patients will be reduced and cardiac efficiency will be increased.

## Introduction

Changes in glucose, fat and protein metabolism are usually observed in patients with diabetes. These metabolic abnormalities can lead to a wide range of long-term effects called diabetes complications. Several studies have shown the directly negative effects of diabetes mellitus on the cardiac muscle (1, 2). In addition, cardiovascular diseases are the main cause of death in diabetic patients, not only due to coronary artery disease and high blood pressure but also due to the direct effects of diabetes complications on the heart, independent of other pathologic factors (3).

However, physical activity affects many physiological systems of the body (4), including the structure and function of the myocardium (5). Studies have shown that the myocardium adapts structurally and efficiently to the type of stimulus provided by physical activity, i.e. endurance or strength (6). Tissue changes resulting from physical activity also take place at the level of gene expression (7) including changes in the biogenesis of mitochondria and myosin heavy chains (MHCs) of the myocardium (8, 9). Mitochondrial biogenesis, or increasing the size and number of mitochondria, is a complex process that requires combined function of different mechanisms and the controlled expression of many genes. PGC-1 which is a cell receptor and facilitates the release of mitochondrial proteins, is the most important regulator of mitochondrial biogenesis. PGC-1 has two, alpha and beta, isoforms, both are involved in this process, but alpha is more important (10).

Studies have shown that other members of this family of transcription coactivators are activated in response to environmental stimuli, such as heat and physical activity. They also play an important role in maintaining glucose homeostasis, lipid homeostasis, energy homeostasis and possibly in pathogenic conditions such as obesity, diabetes, neurodegeneration, and heart diseases (11). Since the heart has a very high energy demand and has basically no energy reserves, it needs to constantly produce great amounts of energy in the form of adenosine triphosphate (ATP) at a high speed to maintain contraction performance and ion homeostasis. Most of the ATP in mitochondria is produced by oxidative phosphorylation, and fatty acids (FA) and carbohydrates are the primary energy substrates. It must be noted that FA account for 50-75% of ATP production in the heart (12, 13). But a diabetic heart cannot completely use glucose due to insulin deficiency, and therefore may be forced to use FA as its energy sources almost exclusively (14). FA metabolism also consumes more oxygen per mole than glucose and thus increases oxidative stress in the heart tissue and reduces cardiac performance (15).

Dichloroacetate (DCA), is imported by cells through the monocarboxylate transporters and mostly a sodiumlinked monocarboxylate transporter also named solute carrier family-5 member 8 (SLC5A8), while access to the mitochondrial matrix is achieved by the mitochondrial pyruvate carrier system (16). Studies have shown that glucose incorporation into glycogen was decreased in diabetic rats when DCA was used to activate pyruvate dehydrogenase (PDH); which was accompanied by an increase in glucose oxidation and a reduction of FA oxidation (beta oxidation) in peripheral tissues of diabetic rats (17). The pyruvate dehydrogenase complex (PDC) is a multifunctional complex in the mitochondrial matrix and has the role of gatekeeper in the tricarboxylic acid cycle (TCA) and oxidative phosphorylation. DCA and some of its derivatives play an important role in this mechanism by activating PDC and regulating cell metabolism in response to diabetes and other conditions that increase the beta oxidation of FA e.g. endurance training (18). However, despite studies done on PGC-1α and its effects on diabetes as well as DCA consumption, findings are still very contradictory and, to the best of our knowledge, a study that can investigate the impact of DCA consumption on *PGC-1α* expression and its relation with aerobic training has not been conducted yet. Given the need to develop therapeutic strategies to prevent or treat diabetes complications, we conducted this study to assess whether DCA consumption after endurance training can reduce the complications of the PGC-1α mechanism in diabetic patients.

## Materials and Methods

In this experimental study, the Ethical guidelines set by Shahid Chamran University of Ahvaz, Iran, was considered during all stages of the experiment (EE/97.24.3.70001/ scu.ac.ir). The present study was designed as a posttestonly with the control groups experiment. In this study, 64 male Wistar rats at 8 weeks of age and weighting 200 ± 12 g were purchased from the Physiology Research Center, Ahvaz Jundishapur University of Medical Sciences, Iran. Rats were kept under the conditions of an even split of 12 hours of light and 12 hours of darkness) at 22 ± 2˚C and 50% humidity, fed with special rat food and water.

After one week of familiarization with the laboratory environment, rats were matched based on weight and were randomly divided into eight groups including healthy control groups (n=7), healthy control group+DCA (n=7), healthy endurance training group (n=7), healthy endurance training group+DCA (n=8), diabetes control group (n=7), diabetes control group+DCA (n=8), diabetes endurance training group (n=8), and diabetes endurance training group+DCA (n=8).

Daily intraperitoneal injections of 50 mg/kg body weight of DCA was used to inhibit PDK4 in the myocardium (19). After 12 hours of food deprivation, induction of diabetes was done by intraperitoneal injection of 50 mg/kg body weight of the STZ solution dissolved in 0.05 M citrate buffer with 4.5 pH (20). The equivalent volume of citrate buffer was also intraperitoneally injected to non-diabetic rats. After 48 hours, with a small lancet cut on the tail vein, a drop of blood was placed on a glucometer strip and the strip was read using a Glucotrend 2glucometer (Roche, Switzerland). Rats whose glucose level was higher than 300 mg/dl were considered diabetic. The rats’ blood sugar levels were measured again at the end of the training program to ensure they had not returned to normal (21).

### Dichloroacetate

DCA was injected to rats intraperitoneally at 50 mg/kg body weight in the form of 24-hour intervals, dissolved in methyl cellulose 400 cP and combined with calcium gluconate (22).

### Endurance training protocol

The protocol was carried out for six weeks (five days/ week). First, training groups were trained for seven days with a treadmill (model LE7800; Harvard Apparatus, France) at a speed of 15 m/minutes for 20 minutes. Then, the duration and speed were gradually increased over the course of six weeks, so that in the final week the speed reached 30 m/ minutes and the training time reached 50 minutes/day, which was equivalent to 75% of the maximum oxygen consumed. Electric shocks were performed on the rats to make them complete the training during the course of the experiment. Control groups were kept in cages untreated during the training period (Table 1) (23).

72 hours after the last training session, 64 rats were anesthetized by intraperitoneal injection of ketamine (90 mg/kg body weight) and Xylazin (90 mg/kg body weight) and the myocardium was immediately removed and frozen in liquid nitrogen and transferred to -80˚C until used for further analysis.

**Table 1:** Training protocol


Week	1(acclimatization)	2	3	4	5	6	7

Speed (m/minutes)	15	20	24	24	28	28	30
Time (minuts)	20	30	30	40	40	50	50


### Real-time quantitative reverse transcription polymerase chain reaction

Isol-RNA was used to extract mRNA. About 100 milligrams of myocardium tissue was ground and homogenized in one milliliter of Isol-RNA Lysis Reagent. Afterwards, the homogeneous product was centrifuged for 10 minutes at 12000 g and 4˚C, the supernatant was removed, and transferred to a new microtube. In the next step, 200 μl of chloroform was added to the separated supernatant and vigorously stirred for 15 seconds. Then, micro tubes were re-centrifuged for 15 minutes at 12000 g and 4˚C. The aqueous phase was removed and 600 μl of isopropyl alcohol was added and centrifuged at 12000 g to extract total RNA. The concentration of RNA and its purity were calculated by controlling the ratio of 260/280 nm OD where values between 1.8 to 2 were defined as acceptable purity. Synthesis of cDNA was carried out using Takara’s cDNA synthesis kit, according to the manufacturer’s instructions. Expression of the desired genes was measured using real-time polymerase chain reaction (PCR) and the results were quantified using the 2-ΔΔCT formula (24). PCR reactions were performed using AMPLIQON RealQ Plus 2x Master Mix Green High ROX. 40 cycles were considered for each cycle of real-time PCR. And the temperatures of each cycle were set at 94˚C for 20 seconds, 60-58˚C for 30 seconds and 72˚C for 30 seconds. GAPDH was used as the reference gene to measure relative gene expression and melting curve analysis was performed to control the specificity of the product. The sequence of the primers used in the study is reported in Table 2.

### Blood analysis

The concentration of PGC-1α in the serum was assessed and quantified using an enzyme-linked immunosorbent assay (ELISA) kit according to the manufacturer’s instructions (Cusabio- EL018425RA-USA). These concentrations were expressed as picograms per milligram of total protein (pg/ml protein). Detection range was in the domain of 125-8000 pg/ml with 31.25 pg/ml sensitivity (Table 3).

### Statistical analysis

Shapiro-Wilk test was used to determine the normality of the data and Levene’s test was used to test the homogeneity of the variances. One-way ANOVA and Tukey’s test were used to determine the difference between the groups’ variables. All statistical analyses were done at a significance level of P<0.05 (SPSS Statistics 22).

## Results

**Fig.1:**
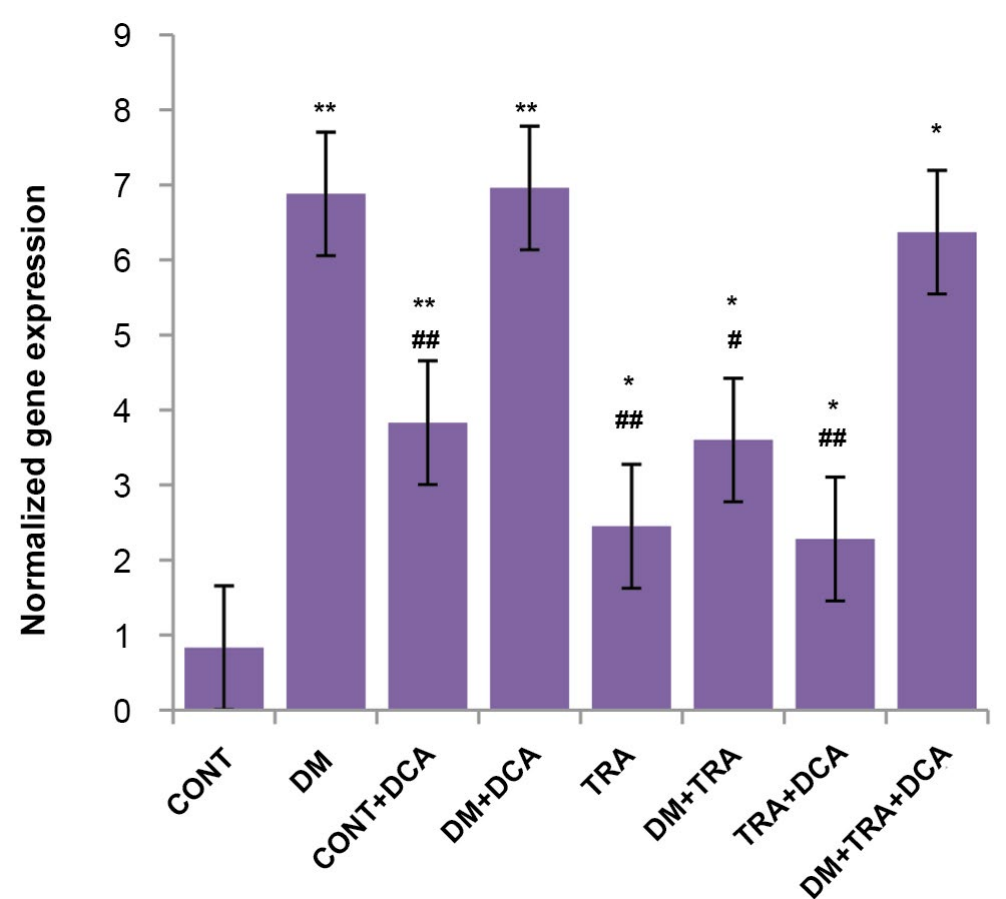
The normalized gene expression of *PDK4* in different groups. From left to right, control group (CONT, n=7); diabetes group (DM, n=7); control+DCA group (CONT+DCA, n=7); diabetes+DCA group (DM+DCA, n=8); healthy group+training group (TRA, n=7); diabetes+training group (DM+TRA, n=8); healthy+training+DCA group (TRA+DCA, n=8); diabetes+training+DCA group (DM+TRA+DCA, n=8). Data are expressed as mean ± SD. *; P<0.05, **; P<0.01 compared with the control group, #; P<0.05, ##; P<0.01 compared with the diabetic group, and DCA; Dichloroacetate.

**Fig.2:**
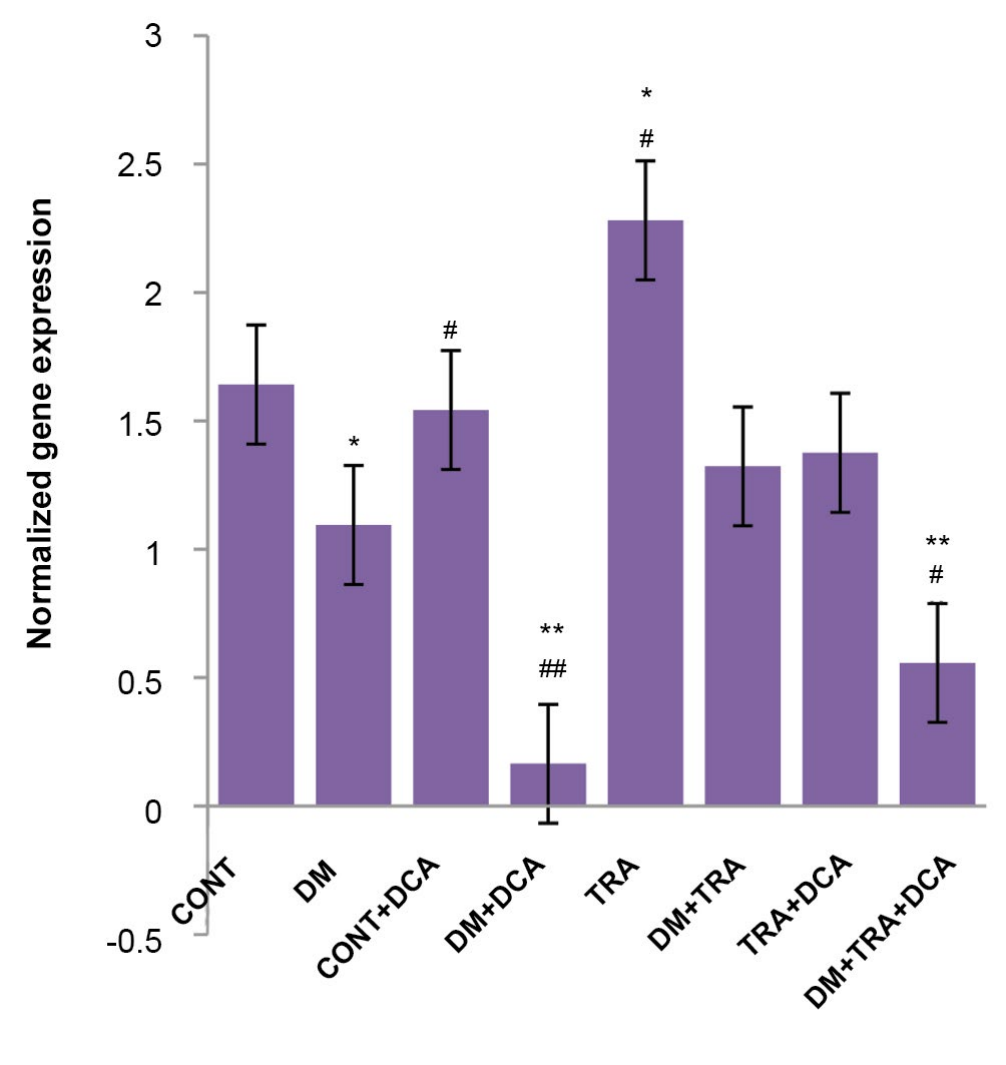
The normalized gene expression of PGC-1α in different groups. From left to right, control group (CONT, n=7); diabetes group (DM, n=7); control+DCA group (CONT+DCA, n=7); diabetes+DCA group (DM+DCA, n=8); healthy group+training group (TRA, n=7); diabetes+training group (DM+TRA, n=8); healthy+training+DCA group (TRA+DCA, n=8); diabetes+training+DCA group (DM+TRA+DCA, n=8). Data are expressed as mean ± SD. *; P<0.05, **; P<0.01 compared with the control group, #; P<0.05, ##; P<0.01 compared with the diabetic group, and DCA; Dichloroacetate.

The results of the study showed that PDK4 gene expression was higher in the endurance training group (P=0.018), diabetes+endurance training group (P=0.008), diabetes+endurance training+DCA group (P=0.001) and endurance training+DCA group (P=0.026) compared to the control group (Fig.1). PGC-1α gene expression in the endurance training group was also higher compared to the control group (P=0.020) but was lower in the diabetes+endurance training+DCA group (P=0.003) and diabetes+DCA group (P=0.001) compared to the control group (Fig.2).

**Table 2:** Mouse-specific primer pairs used for quantitative reverse transcription polymerase chain reaction


Gene	Primer sequence (5ˊ-3ˊ)	Base per	Accession No.

*GAPDH*	F: TGATTCTACCCACGGCAAGTT	21	M17701.1
	R: TGATGGGTTTCCCATTGATGA		
*PGC-1α*	F: TGGAGTCCACGCATGTGAAG	20	NM_013196.1
	R: CGCCAGCTTTAGCCGAATAG		
*PDK4*	F: TATCGACCCCAACTGCGATG	20	NM_053551.1
	R: TGGATTGGTTGGCCTGGAAA		


**Table 3:** Physical characteristics and plasma metabolites of groups


Group	CONT	CONT+DCA	TRA	TRA+DCA	DM	DM+DCA	DM+TRA	DM+TRA+DCA
Variable								

Starting body weight (g)	204 ± 7	207 ± 8	208 ± 9	211 ± 5	205 ± 11	209 ± 6	208 ± 10	209 ± 7
terminal body weight (g)	223 ± 14	198 ± 11	188 ± 10	168 ± 11	216 ± 14	182 ± 12	163 ± 12	149 ± 6
Starting glucose (mg/dl)	104 ± 11	103 ± 6	107 ± 8	108 ± 10	440 ± 81	490 ± 61	412 ± 77	472 ± 58
Terminal glucose (mg/dl)	110 ± 14	111 ± 6	107 ± 8	108 ± 10	407 ± 69	328 ± 52	274 ± 32	178 ± 28
*PGC-1α* (pg/ml)	75.7 ± 12.6	70.1 ± 10.8	59.6 ± 9.1	73.1 ± 14.8	82.3 ± 13.8	70.3 ± 12.3	64.7 ± 11.7	60.5 ± 10.9


Data are presented as mean ± SD. CONT; Control, DCA; Dichloroacetate, TRA; Training, and DM; Diabetes.

### ELISA results

**Fig.3:**
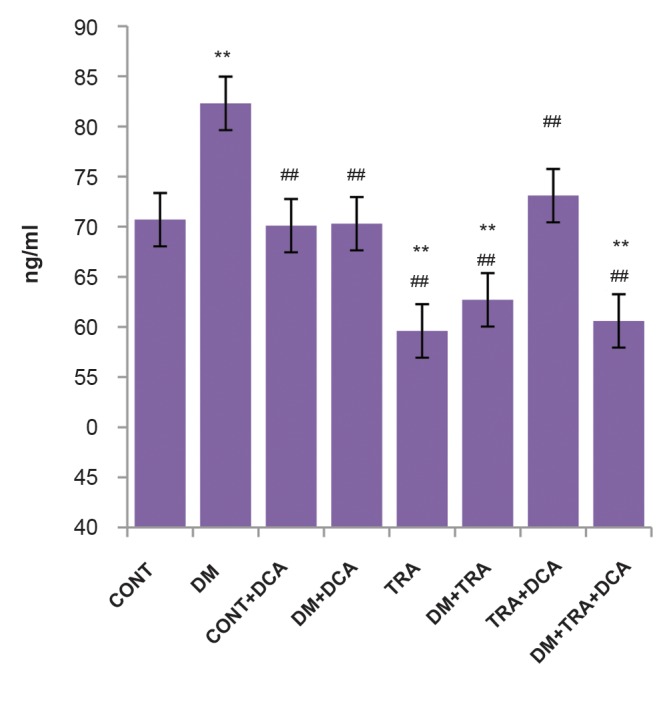
PGC-1α serum levels in different groups. From left to right, control group (CONT, n=7); diabetes group (DM, n=7); control+DCA group (CONT+DCA, n=7); diabetes+DCA group (DM+DCA, n=8); healthy group+training group (TRA, n=7); diabetes+training group (DM+TRA, n=8); healthy+training+DCA group (TRA+DCA, n=8); diabetes+training+DCA group (DM+TRA+DCA, n=8). Data are expressed as mean ± SD. **; P<0.01 compared with the control group, ##; P<0.01 compared with the diabetic group, and DCA; Dichloroacetate.

The results of One-way ANOVA test showed a significant difference in PGC-1α variables (F=72.33, df=7, P=0.001). Also, results of Turkey’s test showed that the mean serum levels of PGC-1α in the diabetic+endurance training group, endurance training group and diabetes+endurance training+DCA was significantly lower than the control group (P≤0.01). But the mean serum levels of PGC-1α in the diabetes group was significantly higher than the control group (P≤0.01, Fig.3).

## Discussion

The purpose of this study was to investigate the effect of *PDK4* inhibition and endurance training on PGC-1α serum levels and gene expression in the cardiac muscle of diabetic rats. The most important results were that after the endurance training, the expression of *PDK4* and *PGC-1α* increased in line with each other. But following inhibition of *PDK4* in the cardiac muscle using DCA, expression of PGC-1α decreased in endurance training+DCA group, the endurance training+diabetes+DCA group, and the diabetes+DCA group. The results also showed that PGC-1α serum levels in the diabetes group was higher than the control group but PGC-1α serum levels in the diabetic+endurance training group, endurance training group and diabetes+endurance training+DCA were lower than the control group. In the present study, DCA as a halogenated carboxylic acid increased the activity of PDC in the animal muscle (25) competitively by controlling PDK2 and PDK4 (26). DCA is known as an activator of PDC (27). Among the more important features of DCA is its ability to lower the blood sugar level in diabetic rats but not cause changes in the blood glucose levels of nondiabetic ones (28).

On the other hand, PGC-1α plays a main role in regulating cellular energy metabolism (29) and by connecting to *PPAR-γ1* and regulating gene expression, it is linked to mitochondrial biogenesis. In addition, it plays an important role in the metabolism of FA and amino acids, secretion of insulin, insulin sensitivity, and obesity. As has been reported, PGC-1α is involved in the pathogenesis of type 2 diabetes mellitus (30). On the other hand, the amount of PGC-1α in aerobic tissues, including the myocardium, is high (31). Studies have shown that the burden of work-induced physical activity on the heart causes a change in the heart myosin heavy chain (MHC) which is similar to what happens in hypertrophy (32). Endurance activities reduce the level of ATP and increase intracellular calcium which activates two pathways, AMP-activated protein kinase (AMPK) and calcium calmodulin/dependent protein kinase (CaMK) (33). The activation of these two pathways leads to an increase in the synthesis of PGC-1α which, by regulating the expression of contractile and enzymatic proteins that participate in the metabolic network, increases the working capacity and also provides the energy needed for increased heart activity. Endurance activity increases the consumption of ATP and a decrease in the amount of ATP activates the AMPK pathway. In this way, *PGC-1α* gene expression in the heart tissue, that is affected by endurance activity, is increased. One of the main actions of the *PGC-1α *gene is mitochondrial biogenesis and thus supply of oxidative enzymes so its increased expression is consistent with increasing aerobic metabolism of the heart (34). Matsuhashi et al. (35) showed in a study that stable activation of the PDH enzyme through PDK4 inhibition by DCA causes excessive CoA production, meaning increased oxidation in the citric acid cycle and leads to histone acetylation which is one of the most important epigenetic processes that occurs to regulate the expression of genes. Inhibition of the PDK4 enzyme following 6 weeks of DCA injection led to increased PDK4 expression at the level of mRNA, which this is a natural response to inhibiting this key enzyme in the metabolism of aerobic energy. *PGC-1α* is involved in the upregulation of the expression of genes regulating FA oxidation in the heart and skeletal muscles (36).

## Conclusion

The results of this study showed that endurance training increased *PDK4* and *PGC-1α* expressions in the cardiac muscle of diabetic rats by inhibiting *PDK4, PGC-1α* expression decreased in the cardiac muscle of diabetic rats. Given that *PGC-1α* plays an important role in mitochondrial biogenesis, it is likely that by controlling PDK4 and subsequently controlling oxidation of FA (beta oxidation) in heart tissue, oxidative stress in the heart tissues of diabetic patients can be reduced and cardiac efficiency increased.
